# Association of the immature platelet fraction with sepsis diagnosis and severity

**DOI:** 10.1038/srep08019

**Published:** 2015-01-26

**Authors:** Rodolfo Monteiro Enz Hubert, Melina Veiga Rodrigues, Bruna Dolci Andreguetto, Thiago M. Santos, Maria de Fátima Pereira Gilberti, Vagner de Castro, Joyce M. Annichino-Bizzacchi, Desanka Dragosavac, Marco Antonio Carvalho-Filho, Erich Vinicius De Paula

**Affiliations:** 1 Faculty of Medical Sciences, University of Campinas, Campinas, SP, Brazil; 2 Hematology and Hemotherapy Center, University of Campinas, Campinas, SP, Brazil

## Abstract

Management of Sepsis would greatly benefit from the incorporation of simple and informative new biomarkers in clinical practice. Ideally, a sepsis biomarker should segregate infected from non-infected patients, provide information about prognosis and organ-specific damage, and be accessible to most healthcare services. The immature platelet fraction (IPF) and immature reticulocyte fraction (IRF) are new analytical parameters of the complete blood count, that have been studied as biomarkers of several inflammatory conditions. Recently, a study performed in critically-ill patients suggested that IPF could be a more accurate sepsis biomarker than C-reactive protein (CRP) and procalcitonin. In this retrospective study we evaluated the performance of IPF and IRF as biomarkers of sepsis diagnosis and severity. 41 patients admitted to two intensive care units were evaluated, 12 of which with severe sepsis or septic shock, and 11 with non-complicated sepsis. Significantly higher IPF levels were observed in patients with severe sepsis/septic shock. IPF correlated with sepsis severity scores and presented the highest diagnostic accuracy for the presence of sepsis of all studied clinical and laboratory parameters. No significant differences were observed in IRF levels. Our results suggest that IPF levels could be used as a biomarker of sepsis diagnosis and severity.

In the last years, the frequency of sepsis is increasing at a rate of nearly 10% per year^1^, probably due to improvements in life expectancy, and the more frequent use of immune suppressive agents and invasive procedures. Despite increasing knowledge about its pathogenesis, mortality rates as high as 30% are still observed, even with best supportive care^1^. Early diagnosis is one of the most important challenges in sepsis management^2^, as delay in sepsis recognition increases sepsis-related mortality. Another important challenge is the heterogeneous nature of sepsis, limiting the effectiveness of a “one-size-fits-all” treatment strategy for these patients^3^. In this context, the discovery of biomarkers capable to identify patients at a higher risk of sepsis complications, or prone to specific sepsis complications, is regarded as a highly relevant field of sepsis research^4^. Currently, procalcitonin and C-reactive protein (CRP) figure among the best studied biomarkers for the diagnosis and monitoring of sepsis^5^,^6^. Nonetheless, barriers such as limited access still preclude their systematic incorporation into sepsis management protocols. Ideally, a sepsis biomarker should be able to: (i) segregate sepsis from other causes of sterile inflammation (SIRS), (ii) allow some kind of risk stratification, and (iii) identify subgroups of patients with specific sepsis complications, enabling target-specific treatments. In addition, an informative biomarker, whose measurement did not depend on complex and high-cost equipments and reagents, would certainly represent an important improvement in sepsis management.

Thrombocytopenia has been recognized as a poor prognostic factor in sepsis for decades based on robust epidemiologic data^7^, and new insights into the cellular pathways of the immune response led to the recognition of platelets as key elements in the host response to an infection^8^,^9^. New hematologic automated analyzers used for evaluation of the complete blood count (CBC), generate a series of advanced analytical parameters that permit a more detailed evaluation of circulating blood cells, including platelets^10^. Parameters such as the immature reticulocyte fraction (IRF) and immature platelet fraction (IPF) provide a more precise evaluation of red blood cell and platelet production, allowing near real-time estimation of erythro and thrombopoiesis^11^,^12^. Diagnostic accuracy studies performed in the last years suggest that both IRF and IPF can provide clinically relevant information about inflammatory activity and disease prognosis^10^,^13^,^14^. In the context of sepsis, a recent study conducted in critically-ill patients suggested that the IPF increases before sepsis becomes clinically manifest, representing a more accurate biomarker than procalcitonin and CRP^15^. The aim of our study was to evaluate the performance of IRF and IPF as biomarkers of sepsis development and sepsis severity.

## Methods

### Study design, patients and clinical data

This was a retrospective observational cross-sectional study aimed to evaluate the association of IRF and IPF levels with the diagnosis of SIRS (systemic inflammatory response syndrome), sepsis and with sepsis severity markers. The study population consisted of all consecutive admissions to two intensive care units (ICU) from a 400-bed academic hospital, occurring from Monday to Friday, during a 30-day period (May 2013). All descriptive data, consisting of demographics, diagnosis, clinical and laboratory data, and sepsis severity scores were obtained from the medical and laboratory records. The study was approved by the local IRB, and was conducted in accordance with the Protocol of Helsinki. All data was de-identified to protect patient confidentiality.

### Sepsis definitions and severity scores

The diagnosis of SIRS, sepsis, severe sepsis, and septic shock in these patients were registered in the medical records by the attending physicians based on classical SIRS and sepsis criteria^30^. Similarly, clinical severity scores were daily assessed by the intensive care staff using the sepsis-related organ failure assessment (SOFA)^31^, and Acute Physiology and Chronic Health Evaluation II (APACHEII)^32^. Sepsis severity scores were retrospectively checked by one investigator, using clinical and laboratory data from the medical records.

### Measurement of IPF and IRF

The IPF and IRF were obtained in an automated hematology analyzer (Sysmex XE5000, Kobe, Japan). These parameters were automatically measured when the first CBC after ICU admission was performed. All IPF and IRF values used in this study were obtained within 24 hours from ICU admission. IPF and IRF were measured in a dedicated reticulocyte/platelet channel of the hematology analyzer by flow cytometry, using a proprietary fluorescent dye containing polymethine and oxazine. This dye penetrates the cell membrane, staining RNA in reticulocytes and in immature (or reticulated) platelets. By analyzing cell volume and fluorescent intensity from these cells, a computer algorithm discriminates (i) red blood cells from reticulocytes; (ii) low, medium and high fluorescent reticulocyte populations; and (iii) platelets with higher RNA content, referred to as immature platelets. The IPF correspond to the fraction (%) of immature platelets from the total platelet population. The IRF corresponds to the fraction (%) of medium and high fluorescence reticulocytes. A local reference ranges for both IPF and IRF had been previously determined from samples of 178 healthy individuals, using the same equipment and analytical protocols.

### Statistical Analysis

Data are expressed as median, range, mean and standard deviation. Continuous variables were compared using the Mann-Whitney test, and categorical variables were compared with the Fisher's exact test. Correlation (Spearman's rank correlation) analysis was performed between sepsis severity scores and CBC parameters. Diagnostic accuracy was estimated using the ROC procedure. A p < 0.05 was considered significant. All analyses were performed using the GraphPad Prism software (GraphPrism Software Inc. San Diego, California, USA).

## Results

### Study population

In total, 41 patients were admitted to both ICUs during the study period. Of these, 23 patients presented criteria for sepsis, and 14 were diagnosed with isolated SIRS. The remaining 4 patients did not present criteria for sepsis or SIRS (they were patients admitted to the ICU for perioperative care). Of the 23 patients with sepsis, 12 presented severe sepsis or septic shock at the time of admission, and 11 had non-complicated sepsis. The median APACHE-II and SOFA of these two subgroups at admission were 15 (range 6–37) and 6 (range 1–17), respectively. Other clinical and demographic characteristics are shown in table 1.

### IPF and IRF in patients with sepsis and SIRS

**Figure 1:**
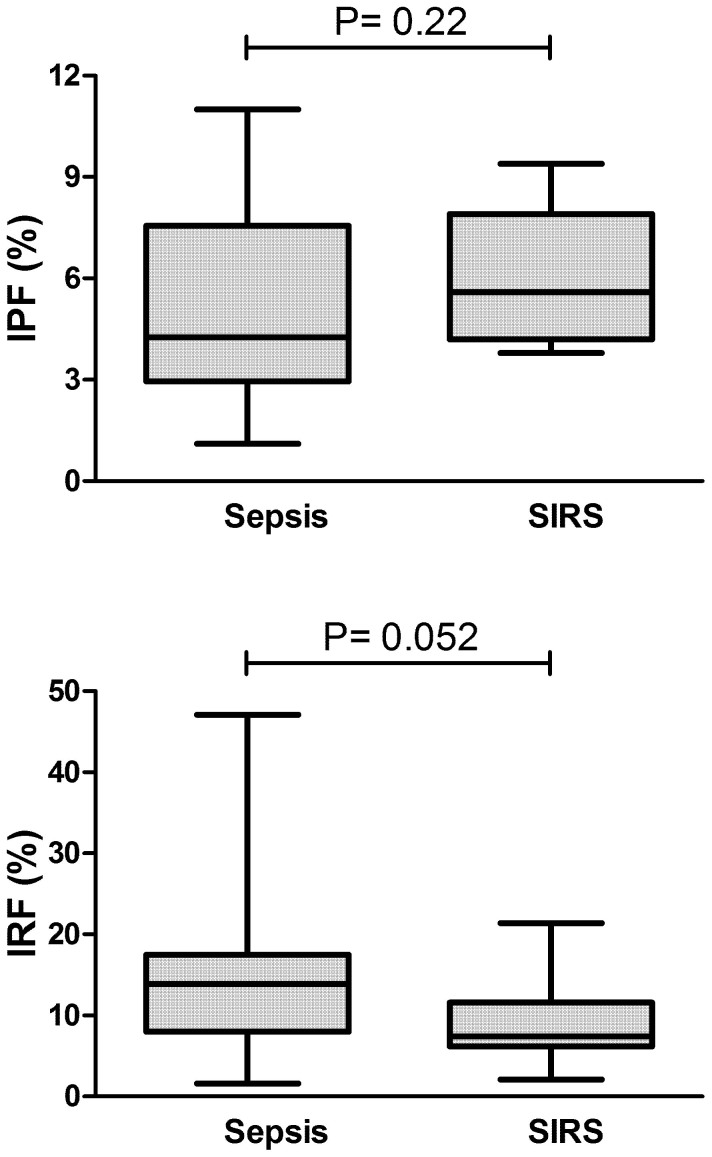
IPF and IRF in sepsis and SIRS. Box-plot showing similar IPF and IRF values in patients with sepsis (n = 23) and SIRS (n = 14). Mann-Whitney test.

We first evaluated whether either the IPF or the IRF could discriminate patients with sepsis from patients with SIRS. As shown in figure 1, IPF was not able to discriminate these two populations. In regard to the IRF, we observed only a trend (P = 0.052) towards lower values in patients with SIRS (figure 1). Of note, our subgroup of SIRS patients consisted mostly of individuals in the immediate post-operative phase of major surgeries, associated with significant bleeding. Since major bleeding, a well-known stimulus for red blood cell and platelet production, can potentially influence IPF and IRF levels, all subsequent analyses considered only the subgroup of patients with sepsis.

### IPF and IRF are higher in sepsis patients than in healthy individuals

**Figure 2:**
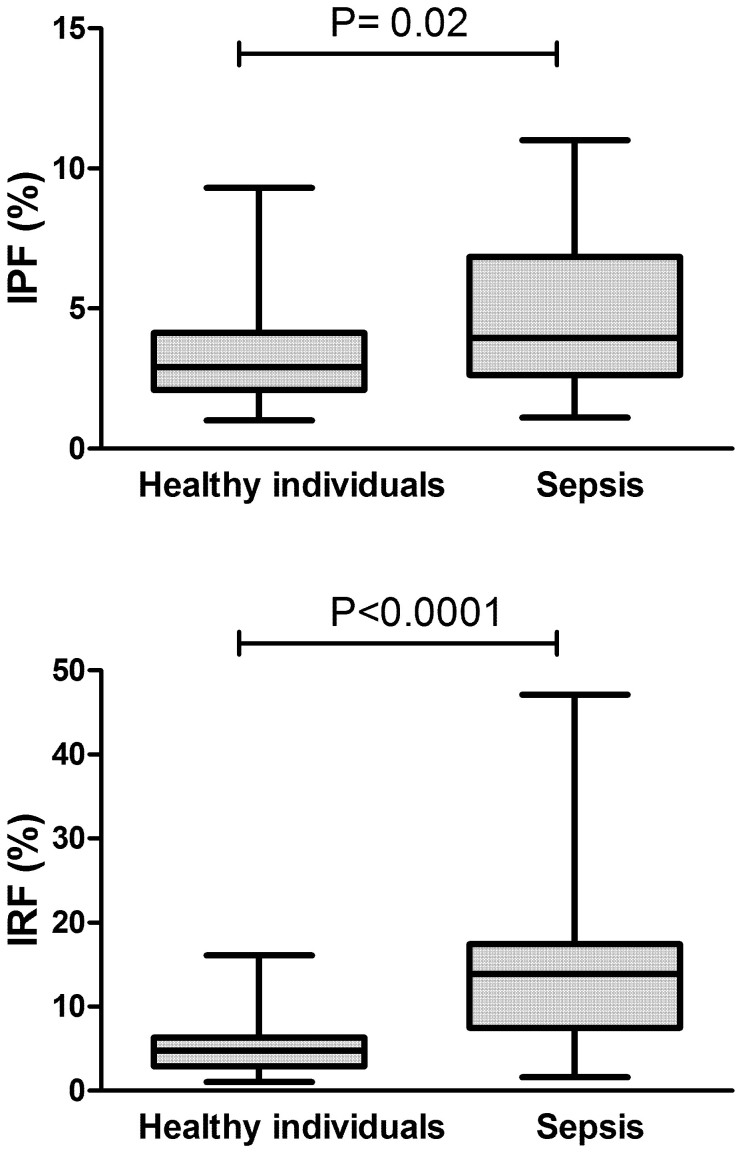
IPF and IRF in sepsis patients compared to healthy individuals. Box-plot showing significantly increased values for IPF and IRF in patients with sepsis (n = 23) compared to healthy individuals (n = 178). Mann-Whitney test.

We next compared IPF and IRF values between patients with sepsis and a population of healthy individuals, used for the determination of our local reference range. As shown in figure 2, despite similar platelet and absolute reticulocyte counts, a significant increase in both IRF and IPF could be observed in our population (n = 23) compared to healthy individuals (n = 178).

### IPF is associated with sepsis severity

**Figure 3:**
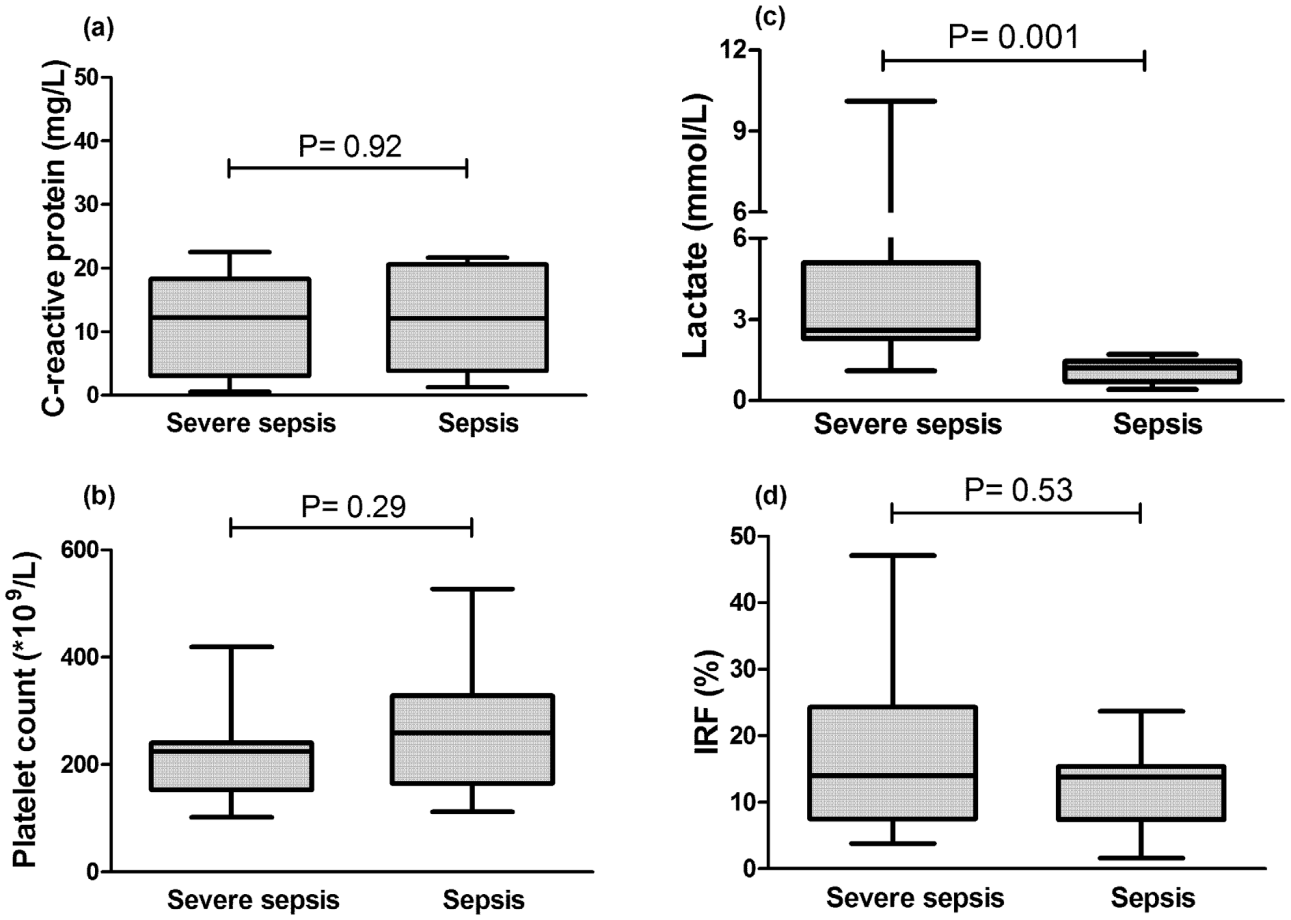
Association of laboratory parameters with sepsis severity. Box-plot showing that neither C-reactive protein (a), platelet count (b), nor IRF (d) presented significant differences between patients with severe sepsis or septic shock (“severe sepsis” group; n = 12), compared to non-complicated sepsis (“sepsis” group; n = 11). As expected, lactate levels were higher in the former group (c). All values obtained at the time of ICU admission. Mann-Whitney test.

**Figure 4:**
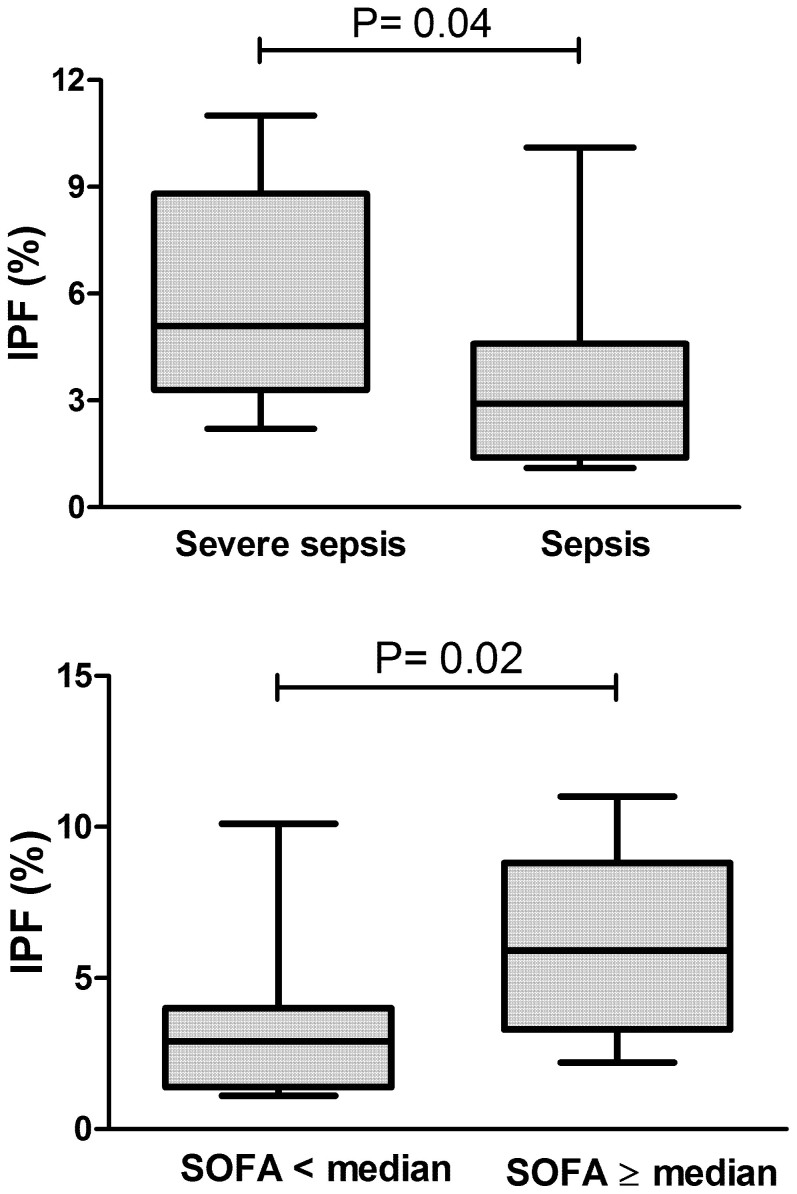
IPF and sepsis severity. Box-plots showing higher IPF in patients with severe sepsis compared to non-complicated sepsis (upper panel). Similar values were obtained when patients were stratified by median SOFA score (lower panel). All values obtained at the time of ICU admission. Mann-Whitney test.

In order to evaluate whether IPF or IRF were associated with sepsis severity, patients with severe sepsis or septic shock (“severe sepsis” group; n = 12) were compared with patients with non-complicated sepsis (“sepsis” group; n = 11). When evaluated at the time of ICU admission, neither C-reactive protein, nor platelet count were able segregate these two subgroups, with only lactate presenting a significant difference between them (Figure 3a–c). IRF values were also similar between these two sepsis subgroups (figure 3d). In contrast, IPF was significantly higher in patients with severe sepsis compared to non-complicated sepsis patients (figure 4a). Similar findings were observed when patients were stratified by the median SOFA score, with patients with a SOFA ≥ 6 presented significantly higher IPF (IPF = 6.2% vs. 2.9%; p = 0.02) (figure 4b).

We also estimated the diagnostic accuracy of several clinical and laboratory parameters evaluated in our patients, for the presence of severe sepsis/septic shock. Within all laboratory parameters, IPF yielded the highest area under the ROC curve (Table 2).

Finally, we investigated if IPF correlated with the SOFA severity score. As previously demonstrated by several authors, a strong inverse correlation between platelet count and the IPF was observed (Rs = −0.70; p < 0.001). No correlation between the SOFA score with platelet count or C-reactive protein could be observed at the time of ICU admission. In contrast, a significant correlation between IPF and the SOFA score could be demonstrated (Rs = 0.50; p = 0.04).

### Association of the IPF with other coagulation laboratory parameters

A secondary objective of our study was to evaluate the association of the IPF with other coagulation laboratory parameters, to explore whether the increase in IPF could represent a marker of coagulation activation during sepsis. No significant correlation could be demonstrated between IPF and the prothrombin time or the APTT (available for all patients with sepsis), nor between the IPF and D-dimer (available in 17 patients with sepsis).

## Discussion

Early recognition is a key goal of sepsis management protocols^2^, since delayed antibiotic therapy increases morbidity and mortality rates of septic shock by 7.6% per hour after the onset of hypotension, and generates significant higher costs to the health care system^16^. For this reason, the search for new biomarkers capable of predicting early sepsis development, as well as the risk of sepsis complications, remains one of the most active areas of sepsis research^17^.

In a recent study, *De Blasi et al*^15^ showed that the IPF was capable to predict the development of sepsis up to 3 days before sepsis become clinically manifest, with IPF values above 4.7% presenting a specificity of 90.0% and a sensitivity of 56.2% for sepsis development. IPF also showed to be the only variable correlated with the development of sepsis^15^. In another study that evaluated the association between high IPF values and the presence of bloodstream infections, *Di Mario et al*^18^ showed that the samples with positive blood cultures had significantly higher mean IPF values (4.86%) than samples with negative blood cultures (1.79%). IPF and IRF are laboratory parameters derived from the CBC in automated hematological analyzers available in most healthcare services^19^. As such, they could represent readily available and low-cost sepsis biomarkers, accessible to several healthcare units.

In our study, IPF and IRF could not discriminate SIRS from sepsis, in contrast to what has been suggested in a previous population of critically-ill patients^15^. Our population of patients with SIRS consisted of patients submitted to complex cardiac surgical procedures, in the immediate post-operative phase. These procedures were associated with extensive trauma, significant bleeding and exposure to extracorporeal membrane oxygenation, all of which could have influenced the kinetics of red blood cells and platelet production. Since the IPF and IRF are very sensitive biomarkers of erythro- and thrombopoiesis^11^,^12^, this characteristic of our population may explain the inability of the IPF to segregate SIRS from sepsis in our study. The lower IRF levels observed in our SIRS patients compared to sepsis could reflect the well-described negative impact of acute inflammation on erythropoiesis^20^,^21^. Accordingly, IRF levels at the same population increased sharply 7 days after ICU admission (data not shown), suggesting that results obtained within 24 hours from major surgeries might be influenced by erythropoietic response to trauma and bleeding. Alternatively, the power of our study to detect a difference in IPF values between sepsis and SIRS might have been lower than necessary. Whatever the reasons might be, critically-ill patients in real-life settings will most certainly include surgical patients, and future studies about the role of IPF as a biomarker of SIRS and sepsis should try to enroll individuals submitted to major surgery, so that results can be generalized to these patients.

On the other hand, when patients with non-complicated sepsis were compared with patients with severe sepsis/septic shock, IPF and lactate were the only significantly different clinical and laboratory parameters. Neither platelet counts, C-reactive protein, nor any other individual laboratory or clinical parameters, including IRF, were different between these two populations at the time of ICU admission. Moreover, within all laboratory parameters, the IPF yielded the highest area under the ROC curve to discriminate severe sepsis/septic shock from non-complicated sepsis. In addition, the IPF also presented a strong correlation with sepsis severity scores such as SOFA and APACHE-II. In this regard, our results confirm the recently described association of the IPF with sepsis severity^15^ in an independent population, supporting the potential of this simple and straightforward CBC parameter as a biomarker of sepsis severity at the time of ICU admission.

The mechanism behind the association of IPF with sepsis and sepsis severity remains to be determined. We believe that higher IPF could be part of the ongoing inflammatory response to sepsis, in which platelets have been shown to play important roles^9^. Accordingly, it has been demonstrated that platelets express many types TLR receptors^8^, which are key regulators of the innate immune response to pathogens. Activation of these receptors leads to the release of IL-1β and formation of neutrophil extracellular traps (NETs)^8^,^22^,^23^, which trap invading pathogens in infection sites. Therefore, higher IPF values in patients with sepsis compared to healthy individuals, as well as its association with sepsis severity might reflect the formation and recruitment of newly formed platelets, as part of a TLR-mediated mechanism triggered by infections. Alternatively, higher IPF levels in sepsis, and especially in patients with severe sepsis, might be a reflex of ongoing disseminated intravascular coagulation (DIC), which results in compensatory platelet production. In fact, IPF has been previously correlated with DIC scores in a cohort of critically-ill patients^24^. In our study, IPF did not correlate with coagulation tests such as prothrombin time, aPTT and D-dimer, but our study was not powered to draw any definite conclusion about this attractive mechanism of IPF increase during sepsis. Although additional studies are necessary to more precisely understand the biological rationale behind the association of IPF and sepsis, IPF should be considered a type 0 biomarker, according to a previously published classification^25^,^26^. Type 0 biomarkers include markers of the natural history of a disease and correlate longitudinally with known clinical indices such as symptoms over the full range of disease states.

Validation of a sepsis biomarker is a stepwise process that must necessarily involve its evaluation in different populations, and in conditions that mimic the so-called “real-life” conditions^27^. Compared to previous reports, our population presents some characteristics that more closely resemble a population in which a sepsis biomarker would be useful such as the inclusion of thrombocytopenic patients, which were excluded from both previous studies that evaluated the IPF in this context^15^,^18^. Examples of discrepancies between preliminary and validation studies despite adequate study design are available for several sepsis biomarkers^28^,^29^, so that confirmatory studies should be regarded as a fundamental part of the initial effort to validate a biomarker. In this context, our observation that IPF is associated with sepsis severity in an independent population adds strength to the recent demonstration of its potential as a sepsis biomarker.

Our study has important limitations that need to be taken into consideration. First, the relative small number of patients may have limited the statistical power to detect a difference in IPF values between sepsis and SIRS. Second, the retrospective nature of our study precluded us to calculate a DIC score, which relies on fibrinogen levels, in our patients. D-dimer levels were available for a subgroup of patients, but a formal evaluation of the association of IPF with DIC was not possible in our study. On the other hand, the unbiased patient selection from two independent ICUs, the adequate characterization of sepsis diagnosis and severity scores, and the inclusion of major surgery and thrombocytopenic patients more closely resembling a real-life scenario in which a sepsis biomarker would be used are important strengths of our study.

## Conclusions

IPF values obtained within 24 hours from ICU admission are higher in patients with sepsis compared to healthy individuals, and correlate with sepsis severity scores. Measurement of the IPF is simple, and can be done as part of a CBC of some automated hematology analyzers. Therefore, our results confirm and extend a recent report of IPF as an informative sepsis biomarker, in an independent and clinically representative population. Larger studies are warranted to define how this readily accessible parameter could be incorporated in sepsis management protocols.

## Author Contributions

R.M.E.H. analyzed data and wrote the manuscript; M.V.R. collected and analyzed clinical data and wrote the manuscript; B.D.A. and T.M.S. collected and analyzed clinical data; M.F.P.G. performed laboratory evaluations; V.C., J.M.A., D.D. and M.A.C. were responsible for patient follow-up, contributed to study design and the study and analyzed data; E.V.D.P. designed the study, and wrote the manuscript. All authors reviewed and approved the manuscript.

## Figures and Tables

**Table 1:** Patient characteristics

	Sepsis (n = 11)	Severe sepsis/ septic shock (n = 12)	P*
Sex (male:female ratio)	7:4	5:7	ns
Age (years) (median, range)	56 (22–85)	59 (38–52)	ns
SOFA – admission (median,range)	**3 (2–9)**	**10 (2–17)**	**0.01**
APACHE-II – admission (median, range)	**12 (6–27)**	**20 (12–37)**	**<0.001**
**SIRS criteria** (mean ± SD)			
Temperature (°C)	37.0 ± 0.8	37.1 ± 0.9	ns
Heart rate (beats per minute)	107.5 ± 18.8	113.5 ± 15.9	ns
Breath rate (per minute)	27.8 ± 14.9	22.7 ± 9.6	ns
White blood cell count (*10^3^/μl)	16.1 ± 10.5	15.9 ± 7.4	ns
Immature forms (%)	10.5 ± 12.6	6.0 ± 9.8	ns
**Additional clinical and laboratory variables** (mean ± SD)			
PaO_2_/FlO_2_ (mmHg)	296.0 ± 115.8	235.6 ± 132.3	ns
Platelet count (*10^9^/l)	271 ± 138	206 ± 102	ns
Mean arterial pressure (mmHg)	97.1 ± 29.7	102.1 ± 11.1	ns
Urine output (l/day)	1.6 ± 5.9	2.1 ± 2.1	ns
Creatinine (mg/dl)	1.7 ± 1.5	1.3 ± 0.8	ns
Bilirrubin (mg/dl)	0.9 ± 0.9	1.8 ± 2.9	ns
C-reactive protein (mg/l)	11.6 ± 8.0	11.6 ± 7.5	ns
Lactate (mmol/l)	**1.1 ± 0.5**	**4.0 ± 2.7**	**<0.001**
D-dimer(μg/ml)	3.1 ± 1.8	3.0 ± 1.5	ns
Prothrombin time (INR)	1.6 ± 1.3	1.5 ± 0.4	ns
aPTT ratio	1.1 ± 0.3	1.2 ± 0.3	ns
**Advanced hematological parameters** (mean ± SD)			
Immature platelet fraction (%)	**3.6 ± 2.6**	**6.2 ± 3.0**	**0.03**
Immature reticulocyte fraction (%)	12.6 ± 6.0	20.6 ± 15.4	ns

*D-dimer levels available for 17 patients. aPTT: activated partial thromboplastin time.

**Table 2:** Estimation of diagnostic accuracy for the presence of severe sepsis/septic shock

Parameter	AUC	95%CI	P
Platelet count	0.66	0.40–0.88	0.28
IPF (%)	**0.76**	**0.56–0.96**	**0.04**
IRF (%)	0.58	0.34–0.83	0.51
C-reactive protein	0.52	0.25–0.79	0.90
APACHE-II	**0.82**	**0.64–1.00**	**0.01**

AUC: area under the ROC curve; CI: confidence interval.
